# Risk of SARS-CoV-2 Infection Among Hospital-Based Healthcare Workers in Thailand at Myanmar Border, 2022

**DOI:** 10.3390/covid5080115

**Published:** 2025-07-25

**Authors:** Narumol Sawanpanyalert, Nuttagarn Chuenchom, Meng-Yu Chen, Peangpim Tantilipikara, Suchin Chunwimaleung, Tussanee Nuankum, Yuthana Samanmit, Brett W. Petersen, James D. Heffelfinger, Emily Bloss, Somsak Thamthitiwat, Woradee Lurchachaiwong

**Affiliations:** 1 Department of Medical Services (DMS), Ministry of Public Health (MoPH), Nonthaburi 11000, Thailand;; 2 Mae Sot General Hospital, Tak 63110, Thailand;; 3 Center for Vaccine Equity, The Task Force for Global Health, Decatur, GA 30030, USA;; 4 Division of Global Health Protection (DGHP), Thailand MoPH—U.S. CDC Collaboration (TUC), Nonthaburi 11000, Thailand;

**Keywords:** risk factor, SARS-CoV-2, healthcare worker, border

## Abstract

**Background:**

This study examined risk factors for syndrome novel coronavirus 2 virus (SARS-CoV-2) infection and self-reported adherence to infection prevention and control (IPC) measures among healthcare workers (HCWs) at a hospital in Thailand near the Myanmar border.

**Methods:**

From March to July 2022, HCWs aged ≥ 18 with COVID-19 exposure at Mae Sot General Hospital completed a questionnaire on IPC adherence, training, and COVID-19 knowledge. Nasopharyngeal samples were collected bi-weekly for SARS-CoV-2 testing. A mobile application was used for real-time monitoring of daily symptoms and exposure risks. Chi-square, Fisher’s exact tests, and log-binomial regression were performed to investigate association.

**Results:**

Out of 289 (96.3%) participants, 27 (9.9%) tested positive for SARS-CoV-2, with cough reported by 85.2% of cases. Nurse assistants (NAs) had a higher risk of infection (adjusted relative risk [aRR] 3.87; 95% CI: 0.96–15.6). Working in inpatient departments (aRR 2.37; 95% CI: 1.09–5.15) and COVID-19 wards (aRR 5.97; 95% CI: 1.32–26.9) was also associated with increased risk. While 81.7% reported consistent hand hygiene, 37% indicated inadequate IPC knowledge.

**Conclusions:**

HCWs, especially NAs and those in high-risk departments, should receive enhanced IPC training. Real-time digital monitoring tools can enhance data collection and HCW safety and are likely to be useful tools for supporting surveillance and data collection efforts.

## 1. Introduction

The global pandemic caused by the severe acute respiratory syndrome novel coronavirus 2 virus (SARS-CoV-2) has resulted in 5.1 million confirmed Coronavirus 2019 (COVID-19) cases reported in Thailand as of June 2025 [1]. Cases arising from cross-border transmission of the virus in persons presenting to Mae Sot General (MSG) Hospital, located six kilometers from the Myanmar border in Thailand, became a concern in late 2020 [2]. To address occupational SARS-CoV-2 infections among health care workers (HCWs) in healthcare settings in Thailand, the Ministry of Public Health (MoPH) has developed guidelines for HCWs to help detect and prevent the transmission of SARS-CoV-2 in this high-risk group in hospitals [3]. In general, testing is recommended for HCWs who work in health facilities (e.g., hospitals, clinics, community health centers), laboratory facilities, and pharmacies if they have any of the following signs and symptoms: history of fever (e.g., temperature > 37.5 °C), cough, runny nose, sore throat, loss of smell, loss of taste, tachypnea, dyspnea, or difficulty breathing [4]. National guidelines require HCWs to follow standard droplet and contact precautions when caring for suspected and confirmed COVID-19 patients, and additional personal protective equipment (PPE), i.e., donning waterproof gowns, gloves, N95 respirators, face shields, goggles, and surgical caps—when performing aerosol-generating procedures, including a collection of nasopharyngeal swabs and oropharyngeal swabs. However, despite clear guidance and testing capacity, there are still no systematic approaches for monitoring and testing of symptomatic and pre-symptomatic patients, which refers to the infected individuals and can spread the virus, but have not yet developed symptoms or asymptomatic HCWs in many hospital settings in Thailand.

To reduce person-to-person transmission of SARS-CoV-2, governments worldwide implemented various prevention strategies, including lockdowns, social and physical distancing, mask mandates, and hygiene practices. These measures significantly impacted daily life, leading to increased healthcare demands, economic challenges, and widespread social disruptions. In this context, application technology became essential for managing and controlling the vast flow of information required for effective pandemic response, including surveillance, communication, and public health coordination [5]. In Thailand, MoPH actively promoted the use of digital tools to enhance public health surveillance, support contact tracing, and strengthen city resilience [6]. Additionally, Thai’s public health professionals also reported that real-time application technologies improved patient monitoring, resource coordination, reduced readmission rates, and increased overall system efficiency [7]. Hence, the digital tools have strong potential to support surveillance, data collection efforts, and timely case detection and response.

During the Omicron wave in February 2022, there was an outbreak with 270 reported cases in Mae Sot District, Tak Province [8], resulting in many infections at MSG Hospital, which placed HCWs at greater risk of infection due to their close contact with COVID-19 patients [9]. From March to July 2022, MSG Hospital implemented syndromic surveillance among HCWs to monitor COVID-19 and detect cases early to reduce transmission in the hospital. We sought to describe the risk of infection and self-reported adherence to general infection prevention and control (IPC) measures among HCWs during the Omicron wave at the MSG Hospital. Leveraging the advantages of digital tools, this study incorporated mobile application digital technology to support data collection, facilitate contact tracing, and assess IPC compliance.

## 2. Materials and Methods

### 2.1. Study Design

A cross-sectional evaluation was conducted from March to July 2022 at MSG Hospital, a major referral hospital in Tak Province, Thailand, to assess the implementation of the IPC program in a large general hospital, utilizing standardized assessment tools developed by the World Health Organization (WHO) [10]. This study aimed to identify gaps in IPC system performance and practice, with a focus on syndromic surveillance of HCWs for COVID-19 symptoms prior to workplace entry, along with routine SARS-CoV-2 detection through real-time reverse transcriptase–polymerase chain reaction (RT-PCR) testing [11]. HCWs aged ≥ 18 years old with direct or potential exposure to COVID-19 were enrolled and completed a baseline questionnaire capturing information on IPC adherence, occupational risk, and vaccination status. A mobile application was utilized to support real-time symptom reporting, data collection, and monitoring, ensuring consistent documentation and analysis of surveillance activities and IPC interventions.

### 2.2. Study Site

This study was conducted at a large healthcare facility under the MoPH, Thailand, where COVID-19 patients have been detected and treated. MSG Hospital is the largest hospital in Tak province, located near the border with Kayin state, Myanmar. The MSG serves as a referral hospital for four community hospitals located along the border, namely, Mae Ramat, Phop Phra, Tha Song Yang, and Umphang.

### 2.3. Population

Eligibility criteria for participation included HCWs at MSG Hospital, aged ≥ 18 years old, who held positions with direct or potential exposure to COVID-19 patients and enrolled during March to July 2022. HCWs involved in providing care for COVID-19 patients, by personnel type, include medical doctors, dentists, pharmacists, nurses working in COVID-19 wards and intensive care units (ICUs), emergency room and acute respiratory infection clinic staff, laboratory technicians, administrative and support staff (e.g., registration staff, cleaners, patient porters, ward clerks).

### 2.4. Participants Enrollment

Eligible HCWs who met the criteria were approached for enrollment following a detailed explanation of this study’s objectives and anticipated outcomes. Those who agreed to participate provided written informed consent prior to enrollment. Minimal information (e.g., age, sex, job title, length of time working in the facility) was recorded on paper for individuals who did not agree to participate, to be able to assess potential bias in non-participants. If HCWs rotated in and out of the hospital to assist in taking care of COVID-19 patients, these HCWs were asked to enroll in the activity and were included for the duration of their stay at the hospital each time. At enrollment, a standardized paper-based questionnaire was utilized to collect HCWs’ information on IPC adherence and training, occupational exposure risk, knowledge, attitudes, and practices related to COVID-19, as well as vaccination history [10].

### 2.5. Study Procedure

HCWs received the instructions and training on how to use a mobile electronic application to report COVID-19 symptoms [9] daily before entering the workplace. COVID-19 symptoms were defined based on national guidelines: history of fever (temperature > 37.5 °C), cough, runny nose, sore throat, loss of smell, loss of taste, tachypnea, dyspnea, or difficulty breathing [4]. A confidential process for screening and reporting symptoms and any potential exposures on arrival for each shift was established. In addition to a daily temperature screen upon entering the building, all HCWs were asked to report via a confidential mobile application on a daily basis (see topic 2.5) whether they have symptoms, the type of symptoms, as well as potential exposures since the previous day. Nasopharyngeal specimens were obtained from HCWs at enrollment and every two weeks, regardless of symptoms, for SARS-CoV-2 real-time reverse transcriptase–polymerase chain reaction (RT-PCR) testing [11].

### 2.6. Development of Electronic Mobile Application to Monitor COVID-19 Risk Management

This system was designed for hospitals to monitor COVID-19 exposure and symptom onset among HCWs in real time, ensuring early detection and timely response. Briefly, all HCWs were required to register in the system by providing information about their working section within the hospital and their professional position. Upon registration, they were added to a dedicated group on the application named “LINE” (LINE｜always at your side), one of the most widely used mobile messaging platforms in Thailand. The LINE application uses its frontend, specifically through the LINE Official Accounts (LINE OAs), with application programming interface (API) and messaging integration, while the backend is powered by Microsoft Structured Query Language (SQL) Server for database management and Microsoft Internet Information Services (IIS) for processing and web hosting. Once HCWs added the LINE OA as a friend, they gained access to menu options tailored to their assigned roles or permissions (e.g., monitoring team or HCWs participants). These menu items link directly to web pages where users can perform their designated tasks within the HCW system. LINE OAs are designed to function seamlessly within the application chat interface, utilizing LINE’s Messaging API and webhook system. This enables automated chatbots to receive user inputs via webhook events and respond with messages, notifications, or targeted broadcasts. This platform also supported two-way communication: HCWs can report symptoms or submit questions through chat, while the monitoring team can respond with direct messages or group-wide broadcasts, ensuring efficient and timely interaction.

**Figure 1.**
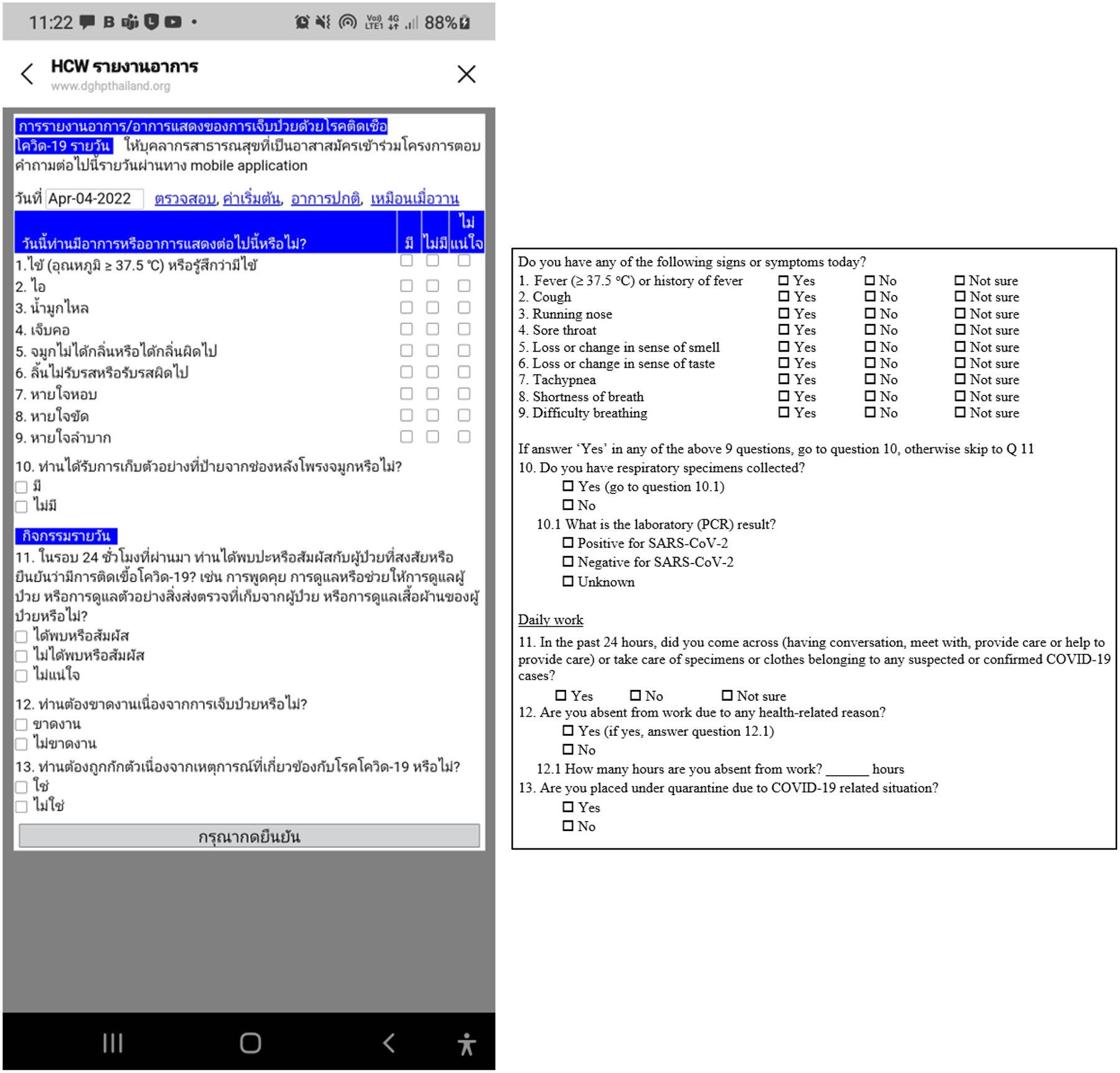
Daily COVID-19 clinical symptoms report window (mobile application): The left figure displays the user interface of the mobile application used for daily reporting of COVID-19-related clinical symptoms. The right figure presents the English-translated version. HCWs are required to respond to Questions 1–10. If they respond “Yes” to at least three of these questions, they must proceed to answer Questions 11–13 before a final case determination is made.

Each day, the monitoring team used a LINE group to automatically send a set of 10 screening questions to all registered HCWs. These questions focused on common COVID-19 symptoms and potential exposure risks. If HCWs responded “yes” to at least 3 symptom-related questions, they were prompted to complete additional questions, questions 11–13, to assess their status (Figure 1). All responses were submitted directly through the LINE application. The answers suggested contact with a confirmed COVID-19 case, and the system automatically notified hospital administrators and supervisors. The affected individual was then promptly guided through appropriate next steps, including quarantine protocols and medical evaluation. All communication is stored as encrypted data in SQL server through LINE OA and secured using the HTTPS protocol. The backend system managed data processing and secure information storage, protected by a firewall.

### 2.7. Statistical Analysis

Descriptive statistics were reported as frequencies and percentages for categorical variables. The association between SARS-CoV-2 positivity and HCWs’ characteristics was assessed using Chi-square/Fisher’s exact test as appropriate. Bivariate and multivariate log binomial regression analyses were performed, and unadjusted and adjusted relative risks (RR) with 95% confidence intervals (CI) were reported. Variables that showed *p* < 0.20 in the bivariate analysis were considered for multivariate analysis. A level of *p* < 0.05 was considered statistically significant. The statistical analysis was conducted using STATA version 15.1 (StataCorp LLC, College Station, TX, USA).

## 3. Results

Mae Sot General Hospital employs a total of 1269 healthcare workers (HCWs), of whom 300 met the eligibility criteria for participation in this study. Among those, 289 (96.3%) provided written informed consent and participated in this active surveillance. The median age of participants was 41 years (interquartile range [IQR] 28–48); 84.1% were female, and 4.2% reported a history of smoking. Participant professions included 52.6% nurses, 8% nurse assistants (NAs), and 2.1% physicians. Overall, 97.2% of participants had received >2 COVID-19 vaccine doses. Of the respondents, 228 (78.9%) were directly involved in patient care, working an average of 40.7 h per week, with an IQR: 40, 56 (Table 1).

The self-reported adherence to IPC measures indicated that 81.7% of HCWs used alcohol-based hand rub or handwashing with soap and water after touching or performing any patient procedure; 78.5% followed hand hygiene practices, and 75.8% wore proper personal protective equipment (PPE). Additionally, 72.2% reported having access to available appropriate PPE, and 64.4% received IPC training. Among 175 HCWs, about 72% reported having physical contact with suspected or confirmed COVID-19 patients in the past 14 days (Table 2). Additional results associated with the knowledge, attitudes, and practices revealed that most HCWs reported strongly agreeing with the importance of wearing surgical masks in public (81.3%) and practicing social distancing (76.8%). Interestingly, 30.1% expressed strong fear of being infected while caring for patients; 27.7% experienced fatigue after taking care of COVID-19 patients, and 11.1% felt that caring for COVID-19 patients caused social stigma. Overall, 37% reported having inadequate IPC knowledge. On the positive note, 97.2% of HCWs practiced good observation by wearing face masks in crowded places (Table 3).

In accordance with the mobile application development, questions related to the COVID-19 symptoms and exposure risks were automatically sent to the participants on a daily basis (Figure 1). Among 274 HCWs who participated in the daily reporting and bi-weekly nasopharyngeal specimen collection, 27 (9.9%) tested positive for SARS-CoV-2 (Table 4). Among those, cough was the most frequent symptom (23 HCWs, 85.2%), followed by sore throat (21 HCWs, 77.8%) and runny nose (20 HCWs, 74.1%), respectively (Table 5). The most frequent symptom combinations were cough and sore throat (20 HCWs, 74.1%), cough and runny nose (19 HCWs, 70.4%), sore throat and runny nose (19 HCWs, 70.4%), and all three symptoms including cough, sore throat, and runny nose were reported by 18 HCWs (66.7%). In the bivariate analysis, being an NA and working in the inpatient department (IPD), COVID-19 ward, and acute respiratory infection clinic were each associated with an increased risk of infection. HCWs who reported caring for or having direct contact with COVID-19 patients did not have a greater risk of COVID-19. Vaccination status, IPC, and COVID trainings were also not associated with the risk of infection. In the multivariable analysis, working in the IPD and COVID-19 ward remained significantly associated with infection, with adjusted RRs of 2.37 (95% CI 1.09–5.15) and 5.97 (95% CI 1.32–26.9), respectively. NAs had an adjusted RR of 3.87 (95% CI 0.96–15.6) for acquiring COVID-19 compared to those in other job categories (e.g., physicians, nurses, and patient caregivers) (Table 4).

## 4. Discussion

Systematic syndromic surveillance and SARS-CoV-2 testing detected COVID-19 in ten percent of HCWs in a large hospital near the border with Myanmar during March-July 2022. The increased aRR of COVID-19 among NAs may have resulted from frequent and prolonged contact with patients [12]. However, it remains unclear whether COVID-19 among HCWs was due primarily to exposures during patient care, cross-transmission between HCWs during other activities, or widespread transmission by asymptomatic patients and HCWs [13]. Nevertheless, previous studies have recommended early detection and isolation of COVID-19 among HCWs to prevent ongoing transmission within hospital settings [14]. Multiple studies have emphasized the critical role of HCWs in both the prevention and transmission of SARS-CoV-2 within healthcare settings. Frontline healthcare workers, especially NAs and those in inpatient or COVID-19 wards, face elevated infection risks due to frequent patient contact and inconsistent access to protective resources [9,15]. Despite the proven effectiveness of IPC practices such as hand hygiene, global compliance remains suboptimal, often due to limited knowledge, high workload, or institutional challenges [16]. Addressing these challenges requires consistent enhancement of IPC training and sustained behavioral reinforcement.

Strict adherence to IPC measures in both healthcare and community settings is vital to prevent disease transmission from symptomatic and asymptomatic persons with COVID-19 [13,17]. Our findings suggest that HCWs’ knowledge and attitudes about COVID-19 (e.g., that caring for COVID-19 patients is stigmatizing, fear of becoming infected), implementation of effective IPC strategies (e.g., standard precautions, social distancing), and adherence to preventive behaviors (e.g., hand hygiene, wearing appropriate PPE) varied widely among HCWs. The symptomatology of SARS-CoV-2 has evolved across its variants, with common symptoms such as fever, cough, and fatigue remaining consistent [18]. Omicron infections often present with upper respiratory tract involvement, such as sore throat and nasal congestion, rather than lower respiratory tract symptoms [19]. In our study during the Omicron wave, the most frequently reported initial symptoms were cough, sore throat, and runny nose, representing the top three clinical manifestations consistent with other findings [20]. Notably, fever was not the top symptom in our cohort, which contrasts with observations elsewhere [18]. Additionally, digital health tools, including real-time mobile applications, have demonstrated value in improving symptom monitoring, data accuracy, and HCW protection during outbreaks [21]. In Thailand, the use of digital tools in the national contact-tracing strategy further underscores their value, with one study highlighting that behavioral factors, such as performance expectancy, privacy concerns, facilitating conditions, and habitual use, significantly influence both the intention to adopt and the actual utilization of these technologies [22].

The integration of the mobile application provided potential benefits since these tools enable real-time symptom tracking, timely communication, and automated alerts, allowing for early detection of potential cases and rapid response to exposure risks. By reducing the need for in-person interactions, digital platforms also support compliance with physical distancing measures. To strengthen the evidence base, future studies should evaluate the effectiveness of these mobile application tools in improving real-time monitoring, IPC compliance, and overall health system resilience in both emergency and routine care settings.

## 5. Limitation

Data in this report were generated from a single hospital, and the sample size was small, leading to insufficient numbers to support the other exposure classifications and the RR analyses. As such, the findings may not be generalizable to all HCWs in Thailand. Nevertheless, identifying potential risk factors for COVID-19 among HCWs can be used to guide efforts to improve prevention and control measures in healthcare settings and support recommendations regarding IPC practices and behaviors.

## 6. Conclusions

This study provides key insights into the knowledge, attitudes, practices, and risks related to COVID-19 among HCWs at a hospital near the Myanmar border. Despite high vaccination rates and adherence to IPC measures, a significant proportion of HCWs tested positive for SARS-CoV-2, with higher risks identified among NAs and those working in IPD and COVID-19 wards. Suboptimal adherence to IPC practices, such as PPE use, hand hygiene, and training, highlights the need for continuous improvement. Although focused on COVID-19, these findings have broader relevance for the prevention and control of other infectious diseases in healthcare settings. Effective IPC strategies are crucial for preventing the transmission of any infectious disease within healthcare environments. These strategies must be adapted to the characteristics of each disease while maintaining robust surveillance and promoting ongoing education to protect both healthcare workers and patients from various infectious threats. Moreover, the mobile digital tools offer a user-friendly and practical platform for daily COVID-19 symptom monitoring among HCWs, enabling real-time alerts, consistent reporting, and timely response to potential cases. The accessibility and ease of integration into daily routines make it a potentially valuable tool for improving surveillance efficiency and supporting healthcare system resilience. Given these potential benefits, future studies recommend evaluating the impact of the mobile digital tools on IPC compliance, outbreak response effectiveness, and overall healthcare preparedness.

## Figures and Tables

**Table 1.** Baseline demographic and job characteristics among health care workers participating in COVID-19 surveillance at MGS hospital from March to July 2022 (*n* = 289).

Characteristics	Number (%)
Demographics	
Age, median (IQR)	41 (28, 48)
Sex	
Male	46 (15.9%)
Female	243 (84.1%)
Vaccination doses	
2	8(2.8%)
More than 2	281 (97.2%)
Job characteristics	
Physician	6 (2.1%)
Nurse	152 (52.6%)
Nurse Assistant	23 (8.0%)
Laboratorian	5 (1.7%)
Patient transporter	6 (2.1%)
Catering staff	7 (2.4%)
Cleaner/housekeeping	10 (3.5%)
Admission, reception, clerk	2 (0.7%)
Administrative staff	7 (2.4%)
Other *	71 (24.6%)
Workstation (*n* = 17, more than one station)	
Inpatient Department (IPD)	96 (33.2%)
Outpatient Department (OPD)	20 (6.9%)
Operating Room	58 (20.1%)
COVID-19 ward	5 (1.7%)
Intensive Care Unit (ICU)	15 (5.2%)
Emergency Room (ER)	21 (7.3%)
Acute respiratory infection (ARI) clinic	2 (0.7%)
Laboratory	5 (1.7%)
Cleaning section	6 (2.1%)
Other	84 (29.1%)
Missing	4 (1.4%)
Time in position, median month (IQR) (*n* = 283)	120 (41.5, 276)
Directly cares for patients	
Yes	228 (78.9%)
No	55 (19.0%)
Missing	6 (2.1%)
Average hours taking care of patients per week (*n* = 228)	40.7
Median (IQR)	40 (40, 56)
Smoking	
Yes	12 (4.2%)
No	277 (5.8%)

* Patient care giver (26), Service staff (13), Public health officer (5), Public health technical officer (8), Driver (3), General administrative officer (2), Traditional Thai medicine doctor (2), Maintenance officer (2), Research assistant of COVID fever project (2), Emerging infectious diseases project staff (1), Medical illustrator (1), Physical therapist (1), Occupational therapist (1), Patient assistant (1), Supply ward staff (1), Nutritionist (1), Security guard (1).

**Table 2.** Baseline self-reported general characteristics related to infection prevention and control (IPC) training and risk activities for health care workers participating in COVID-19 surveillance at MGS hospital during March to July 2022 (*n* = 289).

General Infection Prevention and Control (IPC) Measures				
Characteristics (*n* = 289)	Yes (row %)	No (row %)	Not sure (row %)	Missing (row %)
Training on infection prevention and control (IPC)	186 (64.4%)	50 (17.3%)	37 (12.8%)	16 (5.5%)
Training on COVID-19	145 (50.2%)	84 (29.1%)	41 (14.2%)	19 (6.6%)
Training on performing nasopharyngeal swab	126 (43.6%)	136 (47.1%)	15 (5.2%)	12 (4.2%)
Available appropriate personal protective equipment (PPE) *	210 (72.2%)	32 (11.1%)	34 (11.8%)	13 (4.5%)

General Infection Prevention and Control (IPC) Measures					
Characteristics (*n* = 289)	Always (row %)	Most of the time (row %)	Occasionally (row %)	Rarely (row %)	Missing (row %)
Follow recommended hand hygiene practices	227 (78.5%)	48 (16.6%)	9 (3.1%)	0	5 (1.7%)
Use alcohol-based hand rub or soap and water before touching a patient	213 (73.7%)	53 (18.3%)	10 (3.5%)	2 (0.7%)	11 (3.8%)
Use alcohol-based hand rub or soap and water after touching the patient or performing any patient procedure	236 (81.7%)	36 (12.5%)	5 (1.7%)	1 (0.3%)	11 (3.8%)
Follow IPC standard precautions when in contact with any patient	214 (74.0%)	60 (20.8%)	2 (0.7%)	0	13 (4.5%)
Wear proper PPE as required *	219 (75.8%)	48 (16.6%)	5 (1.7%)	4 (1.4%)	13 (4.5%)

Health Care Worker (HCW) Activities and Exposures to suspected or confirmed COVID-19 patients in the past 14 days				
Characteristics (*n* =289) (%)	Yes (row%)	No (row%)	Not sure (row %)	Missing (row%)
Contact with anyone with an acute respiratory illness	152 (52.6%)	99 (34.3%)	29 (10.0%)	9 (3.1%)
Contact with any suspected ** or confirmed COVID-19 patients.	175 (60.6%)	95 (32.9%)	13 (4.5%)	6 (2.1%)
Physical contact with any suspected or confirmed COVID-19 patients. (*n* = 175)	126 (72%)	31 (17.7%)	18 (10.3%)	0
Characteristics (*n* = 175) (%)	<10 (row%)	10–49 (row %)	≥50 (row%)	Missing (row%)
Number of suspected OR confirmed COVID-19 cases	104 (59.4%)	56 (32.0%)	9 (5.1%)	6 (3.4%)

Frequency of wearing the following PPE during activities with suspected or confirmed COVID-19 patients						
Characteristics (*n* = 126) (%)	Always (row%)	Most of the time (row%)	Occasionally (row%)	Rarely (row%)	Never (row%)	Missing (row%)
Cloth mask	21 (16.7%)	0	0	2 (1.6%)	55 (43.7%)	48 (38.1%)
Surgical mask	108 (85.7%)	3 (2.4%)	1 (0.8%)	0	1 (0.8%)	13 (10.3%)
N-95 respirator	93 (73.8%)	12 (9.5%)	9 (7.1%)	3 (2.4%)	4 (3.2%)	5 (4.0%)
Gloves	94 (74.6%)	12 (9.5%)	4 (3.2%)	3 (2.4%)	2 (1.6%)	11 (8.7%)
Disposable protective medical gown	80 (63.5%)	15 (11.9%)	8 (6.3%)	5 (4.0%)	12 (9.5%)	6 (4.8%)
Cloth gown or disposable non-medical gown	54 (42.9%)	19 (15.1%)	5 (4.0%)	5 (4.0%)	20 (15.9%)	23 (18.3%)
Goggles	30 (23.8%)	7 (5.6%)	4 (4.0%)	5 (4.0%)	54 (42.9%)	26 (20.6%)
Face shield	82 (65.1%)	27 (21.4%)	4 (3.2%)	4 (3.2%)	3 (2.4%)	6 (4.8%)

* Medical mask, N95 mask, face shield, gloves, hospital gown, coveralls, shoe covers.

** The definition of suspected and confirmed cases was defined as following the World Health Organization (WHO) guideline [Public health surveillance for COVID-19: interim guidance (who.int)].

**Table 3.** Baseline knowledge, attitudes, and practices about COVID-19 among health care workers participating in COVID-19 surveillance at MGS hospital from March to July 2022 (*n* = 289).

Attitudes of Health Care Workers Towards COVID-19						
Characteristics	Strongly agree	Agree	Indifferent	Disagree	Strongly disagree	Missing
Afraid of infection while caring for patient	87 (30.1%)	134 (46.4%)	27 (9.3%)	26 (9.0%)	7 (2.4%)	8 (2.8%)
Taking care of COVID-19 patient(s) causes social stigma	32 (11.1%)	74 (25.6%)	55 (19.0%)	90 (31.1%)	30 (10.4%)	8 (2.8%)
Afraid of being placed under quarantine after close contact	36 (12.5%)	110 (38.1%)	34 (11.8%)	79 (27.3%)	21 (7.3%)	9 (3.1%)
Feeling having adequate knowledge about IPC	21 (7.3%)	69 (23.9%)	52 (18.0%)	107 (37%)	31 (10.7%)	9 (3.1%)
Feeling fatigued after taking care of COVID-19 patient	80 (27.7%)	129 (44.6%)	30 (10.4%)	29 (10.9%)	9 (3.1%)	12 (4.2%)
Always wearing mask in public is a good thing to do	235 (81.3%)	43 (14.9%)	1 (0.3%)	1 (0.3%)	1 (0.3%)	8 (2.8%)
Always practicing social distancing is a good thing to do	222 (76.8%)	57 (19.7%)	2 (0.7%)	0	0	8 (2.8%)
Confident that collectively we can cure the disease	184 (63.7%)	75 (26.0%)	19 (6.6%)	1 (0.3%)	0	10 (3.5%)
Patients should disclose their exposure to COVID-19 and their symptoms	203 (70.2%)	68 (23.5%)	8 (2.8%)	1 (0.3%)	0	9 (3.1%)

Practices ofHealthCareWorkersAbout COVID-19					
Characteristics	Always	Most of the time	Occasionally	Rarely	Missing
Maintained quarantine when appropriate	64 (22.1%)	56 (19.4%)	63 (21.8%)	98 (33.9%)	8 (2.8%)
Wears face mask in crowded places	281 (97.2%)	2 (0.7%)	1 (0.3%)	0	5 (1.7%)
Practices hand hygiene at home	175 (60.6%)	80 (27.7%)	26 (9.0%)	2 (0.7%)	6 (2.1%)

**Table 4.** Risk of SARS-CoV-2 infection in a cohort of health care workers (HCWs) participating in this active surveillance in Mae Sot General Hospital, Tak Province, Thailand, March–July 2022 (*n* = 274).

Characteristics	SARS-CoV-2 PCR Positive *n* = 27 (Row %)	Bivariate Analysis		Multivariate Analysis	
		RR (95% CI)	*p*	Adjusted * RR (95% CI)	*p*
Age group					
≥40 y	11 (7.8%)	0.65 (0.31, 1.35)	0.245		
<40 y	16 (12.0%)	Reference			
Sex					
Male	5 (11.4%)	1.19 (0.48, 2.97)	0.712		
Female	22 (9.6%)	Reference			
Job title					
Physician	1 (16.7%)	4.33 (0.53, 35.6)	0.172	0.96 (0.08, 11.1)	0.971
Nurse	15 (10.6%)	2.75 (0.82, 9.20)	0.101	1.86 (0.53, 6.53)	0.331
Nurse assistant	5 (21.7%)	**5.65 (1.46, 21.9)**	**0.012**	3.87 (0.96, 15.6)	0.058
Patient caregiver	3 (12.0%)	3.12 (0.67, 14.5)	0.146	2.96 (0.64, 13.6)	0.163
Other **	3 (3.8%)	Reference			
Work Location Inpatient department					
Yes	15 (17.0%)	**2.64 (1.29, 5.40)**	**0.008**	2.37 (1.09, 5.15)	**0.030**
No	12 (6.5%)	Reference			
Outpatient department					
Yes	2 (11.1%)	1.14 (0.29, 4.43)	0.852		
No	25 (9.8%)	Reference			
Operating room					
Yes	4 (6.8%)	0.63 (0.23, 1.79)	0.382		
No	23 (10.7%)	Reference			
COVID-19 ward					
Yes	2 (40.0%)	**4.30 (1.38, 13.4)**	**0.012**	**5.97 (1.32, 26.9)**	**0.020**
No	25 (9.3%)	Reference			
Intensive care unit					
Yes	2 (13.3%)	1.38 (0.36, 5.29)	0.637		
No	25 (9.7%)	Reference			
Emergency room					
Yes	3 (13.6%)	1.43 (0.47, 4.38)	0.529		
No	24 (9.5%)	Reference			
Acute respiratory infection clinic					
Yes	1 (50.0%)	5.23 (1.25, 21.9)	0.024		
No	26 (9.6%)	Reference			
Laboratory					
Yes	0 (0%)	NA	-		
No	27 (10.0%)				
Cleaning section					
Yes	0 (0%)	NA	-		
No	27 (10.1%)				
Other ***					
Yes	6 (7.4%)	0.68 (0.29, 1.62)	0.386		
No	21 (10.9%)	Reference			
Work Location					
≥2 units/departments	3 (17.6%)	1.89 (0.63, 5.65)	0.255		
1 unit/department	24 (9.3%)	Reference			
Direct care for patients (*n* = 269)					
Yes	23 (10.6)	1.35 (0.48, 3.72)	0.568		
No	4 (7.8%)	Reference			
Direct contact with patients (*n* = 264)					
Yes	21 (10.3%)	1.26 (0.49, 3.20)	0.625		
No	5 (8.2%)	Reference			
Existing medical conditions					
Yes	7 (8.9%)	0.86 (0.38, 1.96)	0.727		
No	20 (10.3%)	Reference			
*Existing Medical Conditions*					
Obesity					
Yes	3 (15.1%)	1.59 (0.52, 4.81)	0.415		
No	24 (9.4%)				
Hypertension					
Yes	3 (10.0%)	1.02 (0.33, 3.18)	0.977		
No	24 (9.8%)				
Diabetes					
Yes	1 (16.7%)	1.72 (0.28, 10.7)	0.561		
No	26 (9.7%)				
Cancer					
Yes	0 (0.0%)	NA	-		
No	27 (10.0%)				
Cardiovascular disease					
Yes	0 (0.0%)	NA	-		
No	27 (10.0%)				
Smoking					
Yes	0 (0.0%)	NA	-		
No	27 (10.3%)				
COVID-19 vaccination					
2 doses	1 (14.3%)	1.47 (0.23, 9.34)	0.685		
>2 doses	26 (9.7%)	Reference			
Infection, prevention, and control training (*n* = 260)					
Yes	16 (8.9%)	0.80 (0.37, 1.74)	0.581		
No/Not sure	9 (11.1%)	Reference			
Training on COVID-19 (*n* = 257)					
Yes	12 (8.6%)	0.84 (0.39, 1.79)	0.644		
No/Not sure	12 (10.3%)	Reference			

RR: relative risk, CI: confidence interval.

* Adjusted for variables with *p* < 0.20 in the bivariate analysis, including job title, workstation in the inpatient department, and COVID-19 ward. The acute respiratory infection clinic was excluded from the model due to small sample size, and the estimate could not be converged.

** Including laboratorian, patient transporter, catering staff, cleaner, administrative staff, admission/reception/ward clerk, others but not patient caregiver.

*** Including anesthesia, center of medical equipment, central kitchen, central sterile supply department, chemotherapy, community health nursing, dental, disease control and epidemiology, driver, emerging infectious project, finance and accounting, health promotion educator, hemodialysis department, hospital director, infectious control nurse, labor room, maintenance, medical rehabilitation, medicine, nutritional science, occupational therapy, physical therapy, psychiatry and drug dependence, social medicine, special care, Thai traditional medicine, translator.

**Table 5.** Report * signed, symptoms and exposure history among PCR positive with SARS-CoV-2 infection in a cohort of health care workers (HCWs) participating in this active surveillance in Mae Sot General Hospital, Tak Province, Thailand, March–July 2022 (*n* = 27).

Characteristics	SARS-CoV-2 PCR Positive (%)
Cough	23 (85.2)
Sore throat	21 (77.8)
Runny nose	20 (74.1)
Fever	12 (44.4)
Change or loss of smell	4 (14.8)
Change or loss of taste	3 (11.1)
Short of breath	2 (7.4)
Difficulty breathing	2 (7.4)
Tachypnea	1 (3.7)

* Daily reporting: within 3 days before and after the sampling date of PCR positive samples.

## Data Availability

The data presented in this study are available on request from the corresponding author. The data are not publicly available due to ethical restrictions.
